# Nonsurgical Management of Extensive Internal Root Resorption: A 48‐Month Follow‐Up Case Report

**DOI:** 10.1155/crid/4482522

**Published:** 2026-10-22

**Authors:** Hamdi Alperen Yalcin, Aybike Sayar

**Affiliations:** ^1^ Department of Endodontics Faculty of Dentistry Aksaray University Aksaray Turkey aksaray.edu.tr; ^2^ Department of Pediatric Dentistry Faculty of Dentistry Aksaray University Aksaray Turkey aksaray.edu.tr

**Keywords:** case report, cone-beam computed tomography, internal root resorption, mineral trioxide aggregate, root canal obturation

## Abstract

A 21‐year‐old male presented with a nonvital right maxillary central incisor with percussion sensitivity and mild mobility. Cone‐beam computed tomography (CBCT) confirmed extensive internal root resorption (IRR) involving nearly the entire root, with a buccal cortical bone defect but no perforation of the root. Nonsurgical treatment was completed in three sessions using chemomechanical preparation with ProTaper Gold and 2.5% sodium hypochlorite irrigation at each session, with an interim calcium hydroxide dressing between sessions. The calcium hydroxide was removed using 17% EDTA with EDDY sonic activation, followed by a final saline rinse. Obturation was performed using a hybrid technique: MTA Angelus in the resorptive defect and an epoxy resin sealer with warm vertical gutta‐percha condensation coronally. Clinical and radiographic follow‐ups at 6, 12, and 48 months demonstrated resolution of clinical symptoms, periapical healing, and no further progression of resorption on periapical radiographs. A follow‐up CBCT at 48 months showed healing of the buccal cortical bone defect. In this case, nonsurgical orthograde treatment, combining CBCT‐guided diagnosis, chemomechanical debridement, and hybrid bioceramic–resin obturation, was associated with clinical and radiographic healing, suggesting that a nonsurgical orthograde approach may be a feasible tooth‐preserving option for selected cases of extensive nonperforating IRR.

## 1. Introduction

Internal root resorption (IRR) is an uncommon pathological condition characterized by the progressive loss of intraradicular dentin due to clastic cell activity within the pulp space. This process is typically associated with chronic pulpal inflammation and may remain asymptomatic for extended periods, often being detected incidentally during routine radiographic examination [1]. If left untreated, the resorptive process may progress, leading to weakening of the tooth structure, root perforation, and eventual tooth loss.

Although the exact etiology remains unclear, trauma, pulpal infection, and previous restorative or endodontic procedures have been suggested as predisposing factors [2]. Early diagnosis is critical for a favorable prognosis; however, the accurate identification of internal resorption may be challenging, particularly in its initial stages. Conventional radiographic techniques provide limited two‐dimensional information, which may hinder the differentiation between internal and external resorptive defects in the tooth structure. In this context, cone‐beam computed tomography (CBCT) has been shown to significantly improve diagnostic accuracy by providing three‐dimensional visualization of the lesion and its extent [3].

The primary objective in the management of IRR is to eliminate inflamed pulp tissue, arrest the resorptive process, and preserve the structural integrity of the tooth. However, the irregular morphology of resorptive defects presents a major challenge for effective debridement and disinfection. The recent literature continues to emphasize that conventional syringe irrigation is insufficient in complex canal anatomies, and adjunctive irrigant activation techniques are required to enhance disinfection. Among these, irrigant activation techniques have been investigated for improving debris and tissue removal in complex root canal anatomies. Sonic and ultrasonic activation may enhance irrigant penetration into anatomical irregularities and isthmuses of the root canal system [4]. Activation systems may also improve cleaning efficacy in anatomically complex areas that are difficult to access with conventional irrigation alone [5, 6].

In addition, advances in biomaterials have improved treatment outcomes. Calcium silicate–based materials, including mineral trioxide aggregate (MTA) and bioceramic sealers, have gained widespread acceptance owing to their favorable sealing ability, biocompatibility, and capacity to promote hard tissue formation. These materials are considered useful in cases with resorptive defects and perforations, as they may adapt to irregular canal walls and support tissue healing [7, 8].

The management of IRR can involve both surgical and nonsurgical (orthograde) approaches, with the latter becoming increasingly prioritized owing to recent advancements in endodontic technologies [9]. A nonsurgical approach is preferred when the resorptive defect is accessible through the root canal system and can be adequately disinfected and sealed using bioactive materials [10].

The present report describes a case of extensive IRR managed with a nonsurgical approach, using sonic activation for removal of the calcium hydroxide dressing and a bioceramic material for obturation, with a 48‐month follow‐up, and discusses it in the context of the relevant literature.

## 2. Case Presentation

### 2.1. Patient Information

A 21‐year‐old male patient presented to our clinic. The patient reported a history of severe pain in the right maxillary central incisor, which had an old, discolored restoration. He had previously attended an emergency department, where antibiotics were prescribed without dental intervention, and extraction followed by implant placement was advised by some of the practitioners he consulted. Approximately 2 weeks before presentation, he visited a private clinic because of swelling in the right maxillary central incisor region; there, an access cavity was prepared and the tooth was sealed with a temporary filling. Because of the complexity of the case, he was referred to our clinic. At presentation, he reported no spontaneous pain but described an unusual numb sensation in the tooth and felt that it was unusable. The patient had no known history of dental trauma or systemic illnesses.

### 2.2. Clinical Findings

Informed consent for the procedure was obtained from the patient, and explicit written consent for the publication of this case report was also obtained. A clinical examination revealed that an access cavity had been created in the affected tooth and subsequently sealed with a temporary filling material. The tooth did not respond to pulp sensibility testing, and palpation was negative; in addition, percussion sensitivity and mild mobility were detected.

### 2.3. Timeline

The timeline of the episode of care is presented in Table 1.

**Table 1:** Timeline of the episode of care.

Time point	Events and findings
Approximately 3 weeks before referral	The patient experienced severe pain in the right maxillary central incisor, which had an old, discolored restoration. The patient subsequently visited an emergency department, where antibiotics were prescribed, but no dental intervention was performed; the attending clinicians there recommended extraction followed by implant placement.
Approximately 2 weeks before referral	The patient initially presented to a private dental clinic with a complaint of swelling. An access cavity was prepared, and the tooth was temporarily sealed. Because of the complexity of the case, the patient was subsequently referred to our clinic for further evaluation and treatment.
Day 0 (first visit)	Session 1: no spontaneous pain; unusual numb sensation reported. Percussion sensitivity, mild mobility, no response to pulp sensibility testing. Periapical radiograph and CBCT: extensive IRR, buccal cortical bone defect, no root perforation. Session 1: canal preparation (ProTaper Gold to F3) with 2.5% NaOCl, saline rinse, calcium hydroxide dressing.
Week 3	Session 2: symptoms had subsided; calcium hydroxide removed with 17% EDTA and EDDY sonic activation, saline rinse, 2.5% NaOCl; calcium hydroxide dressing renewed.
Week 6	Session 3: calcium hydroxide removed with 17% EDTA and EDDY sonic activation, saline, 2.5% NaOCl, final saline rinse; obturation with MTA Angelus in the resorptive defect and epoxy resin sealer with warm vertical gutta‐percha coronally; coronal composite restoration completed.
6, 12, and 48 months after treatment	Clinical and periapical radiographic follow‐up: no pain, tenderness to percussion, swelling, sinus tract, or mobility; periapical healing; no radiographic progression of the resorption. Follow‐up CBCT at 48 months: healing of the buccal cortical bone defect.

### 2.4. Diagnostic Assessment

Radiographic examination revealed a radiolucent area in the apical region and evidence of IRR (Figure 1). CBCT imaging was performed to provide a three‐dimensional assessment of resorption, its extent, and to locate any potential perforation. CBCT evaluation revealed a buccal cortical bone defect in the middle third of the right maxillary central incisor, with no evidence of root perforation; moreover, a resorptive area involving nearly the entire root was detected (Figures 2 and 3).

**Figure 1:**
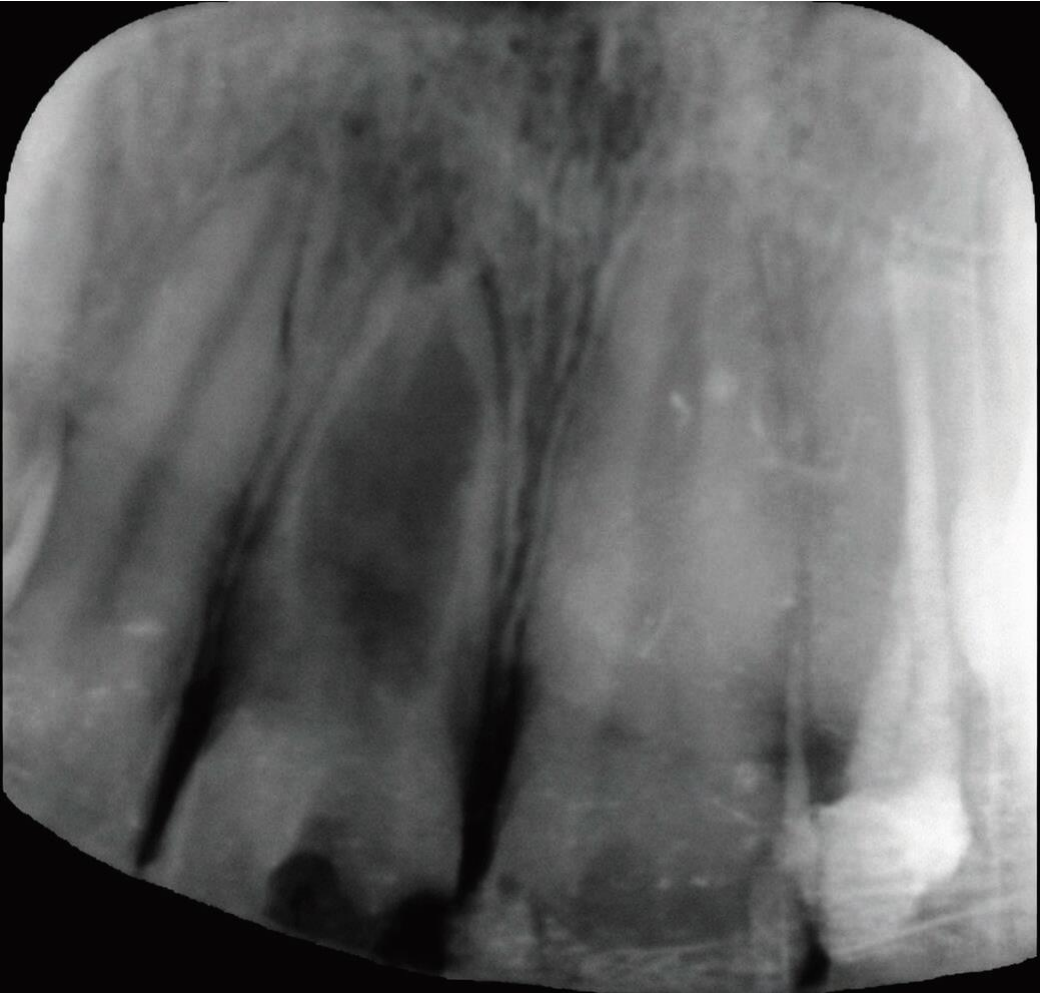
Preoperative periapical radiograph.

**Figure 2:**
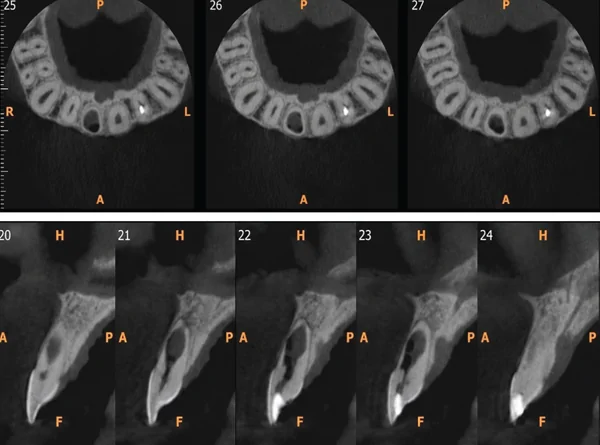
Preoperative CBCT imaging shows the spread of internal resorption inside the root.

**Figure 3:**
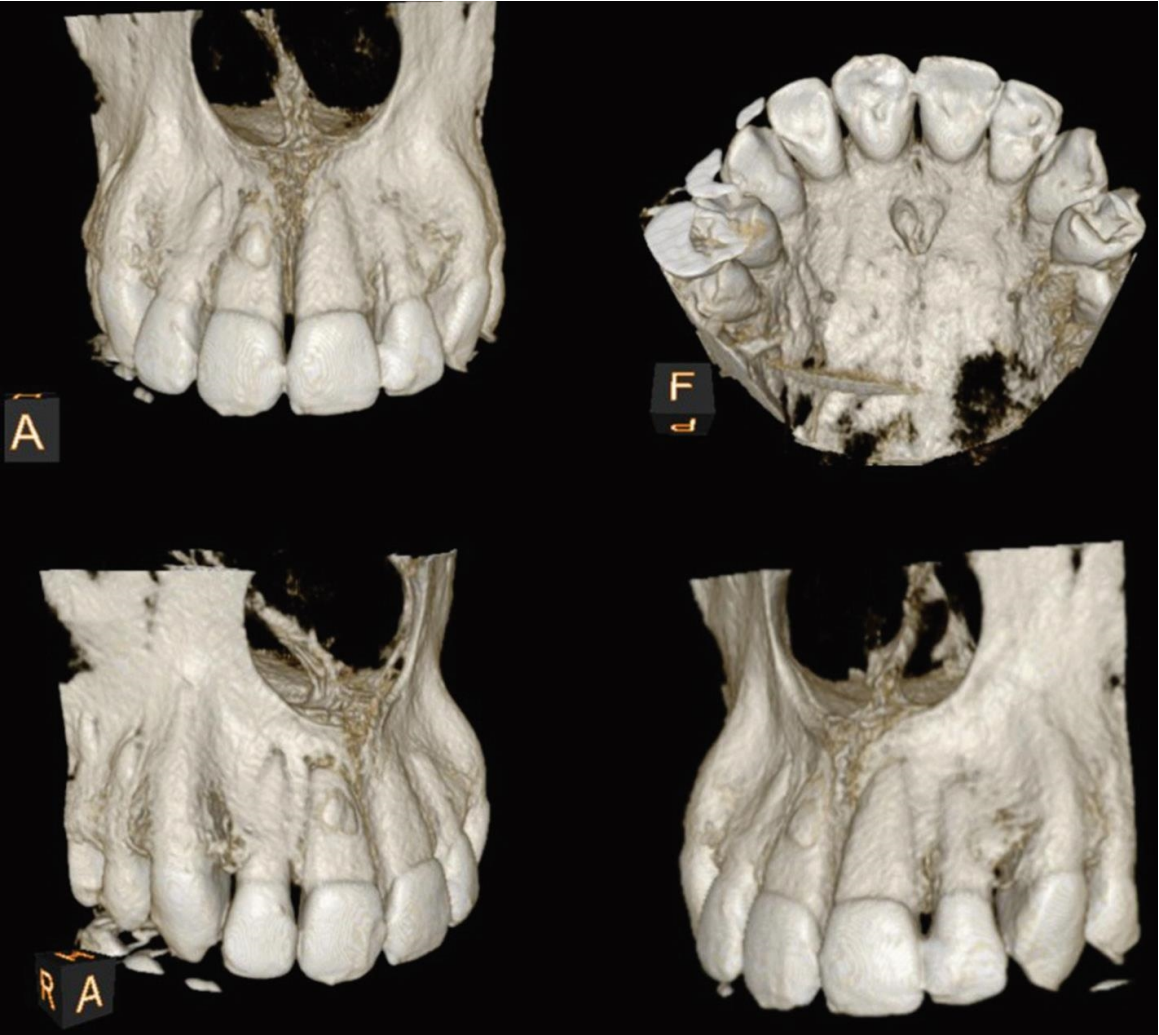
Preoperative 3D CBCT images show buccal cortical bone destruction.

### 2.5. Differential Diagnosis

External inflammatory root resorption (EIRR) should be considered in the differential diagnosis of IRR. EIRR typically occurs as a result of trauma, infection, or damage to the cementum, leading to clastic activity on the external root surfaces. Radiographically, it presents as irregular radiolucent areas along the root surface, often associated with the loss of lamina dura and adjacent bone resorption [1, 2].

In contrast, IRR is characterized by a well‐defined, symmetrical enlargement of the root canal space, with the original canal outline remaining continuous. In the present case, the radiographic appearance demonstrated uniform enlargement of the canal space without disruption of the external root surface, supporting the diagnosis of IRR rather than an external process [1].

External cervical resorption may also mimic internal resorption on radiographic images. However, cervical resorption typically originates from the cervical region of the tooth and presents as an irregular radiolucency with poorly defined margins. Additionally, the root canal outline usually remains intact and can be traced through lesions. In the present case, the lesion was centered within the root canal space, which ruled out cervical resorption [1–3].

### 2.6. Therapeutic Intervention

#### 2.6.1. First Session

Treatment was initiated under local anesthesia (Maxicaine, 4% articaine, 1:100,000 epinephrine, Vem İlaç, Ankara, Turkey). Following the opening of the tooth access cavity (Figure 4), the canal length was determined as 20 mm using a #30 K‐file (M‐Access, Dentsply Maillefer, Ballaigues, Switzerland) and an electronic apex locator (Woodpex III, Woodpecker, Guilin, China) under rubber dam isolation and confirmed radiographically. After the apical portion was widened with a #30 K‐file according to the manufacturer′s recommendations, the canal was prepared from S1 to F3 using a crown‐down technique with the ProTaper Gold (Dentsply Maillefer, Ballaigues, Switzerland) file system. In addition, 2.5% sodium hypochlorite (NaOCl) irrigation was performed between each file; a reduced concentration was chosen to minimize the risk of extrusion of the irrigant into the periradicular tissues because of the extensive resorptive defect (see Section 3). Granulation tissue in the pulp chamber was removed. At the end of the session, irrigation was performed using saline, and calcium hydroxide (CalciPlus, IMICRYL, Turkey) was placed in the root canal (Figure 5). The patient was recalled after 3 weeks.

**Figure 4:**
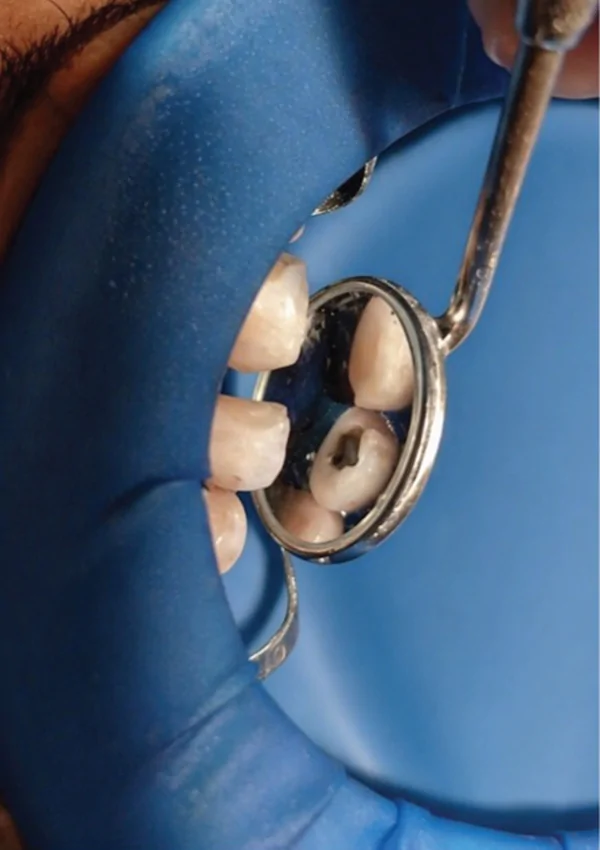
Access cavity image.

**Figure 5:**
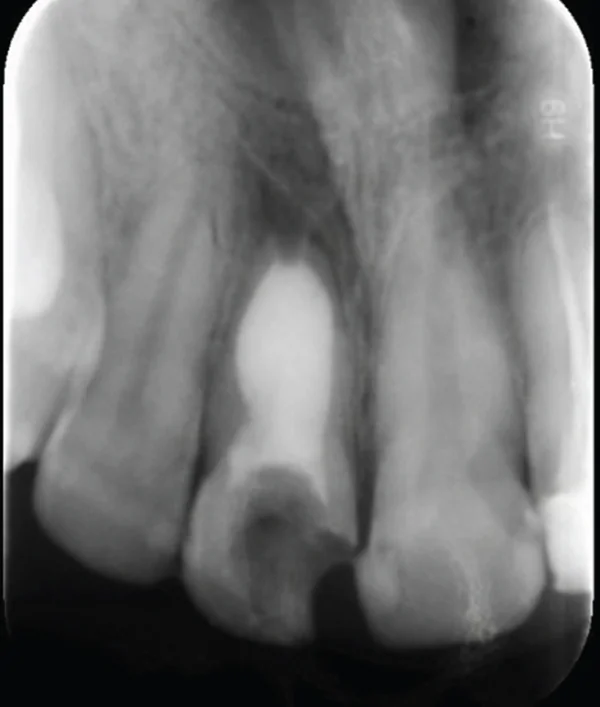
Periapical radiograph showing calcium hydroxide placement.

#### 2.6.2. Second Session

By the time of this appointment, the patient′s symptoms had subsided. The tooth cavity was reopened, and the irrigation protocol was performed as follows: Using a 20‐G Navi‐Tip irrigation needle (Ultradent Products Inc., South Jordan, Utah, United States), 2 mL of 17% ethylenediaminetetraacetic acid (EDTA) was applied to the root canals for 20 s, followed by activation for 20 s. This sequence was repeated eight times, with a total of 16 mL of 17% EDTA applied. Activation was performed with an EDDY (VDW, Munich, Germany) flexible polyamide tip (25/0.4) providing sonic activation (Figure 6). Following EDTA activation, the canal was rinsed with saline and then irrigated with 2.5% NaOCl. Calcium hydroxide was renewed, and the patient was recalled after 3 weeks.

**Figure 6:**
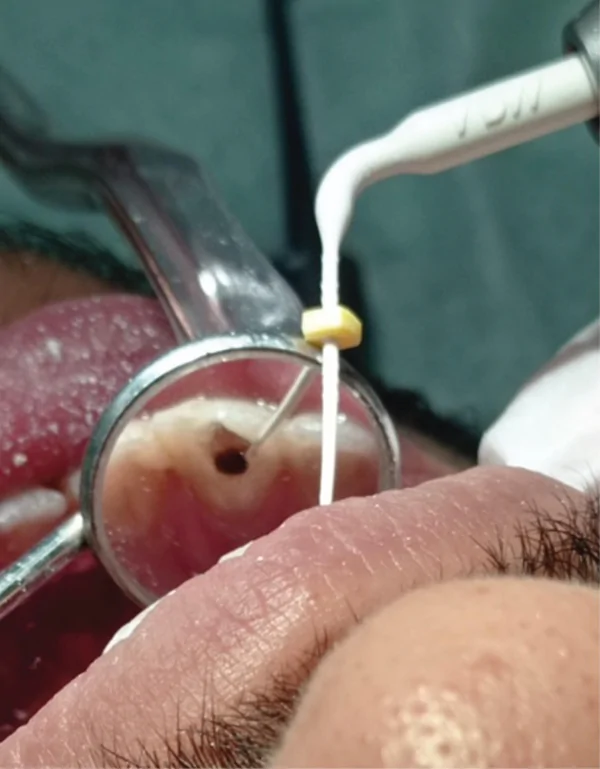
Irrigant activation with EDDY.

#### 2.6.3. Third Session

At the third session, the calcium hydroxide dressing was removed using 17% EDTA with EDDY sonic activation, following the same protocol as in the second session. The canal was then rinsed with saline, irrigated with 2.5% NaOCl, and finally rinsed again with saline.

The root canal space was dried using sterile paper cones. Root canal filling was performed using MTA Angelus (Angelus, Londrina, Paraná, Brazil) bioceramic repair material from the apex to the thin root dentin area in the middle third. In this context, MTA was condensed in the canal using a plugger (EndoArt, Inci Dental, Istanbul, Turkey). The initial setting of MTA was completed within approximately 10–15 min [11]. Subsequently, an epoxy resin–based root canal sealer (ADSEAL; Meta Biomed, Korea) was placed in the coronal portion using a sterile paper point and activated with an EDDY sonic device. The coronal portion of the root canal was filled up to the enamel‐cementum junction using the hot vertical condensation technique (elements free Backfill, Kerr Endodontics, Orange, California, United States) (Figure 7A,B). After the canal orifice was sealed with a flowable composite (G‐ænial Universal Flo, GC Corporation, Tokyo, Japan), the coronal restoration was completed in the same (third) session using a single‐step self‐etch adhesive (G‐Bond, GC Corporation, Tokyo, Japan) and a nanohybrid composite resin (G‐ænial Anterior, GC Corporation, Tokyo, Japan). The composite was applied incrementally, and each 2‐mm layer was light‐cured for 20 s.

**Figure 7:**
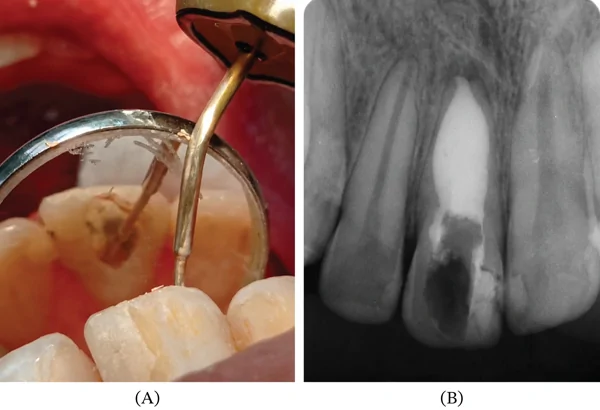
(A,B) Warm vertical compaction of gutta‐percha and postoperative periapical radiograph.

### 2.7. Follow‐Up and Outcomes

The patient was followed up at 6, 12, and 48 months after treatment. At each follow‐up visit, clinical examination revealed no pain, tenderness to percussion, swelling, or sinus tract formation. The tooth remained functional, with no signs of mobility or periodontal involvement. Radiographic evaluation demonstrated healing of the periapical lesion and no evidence of further progression of the resorptive process. No signs of reinfection were observed (Figure 8A–C). At the 48‐month visit, the tooth was asymptomatic, and a slight darkening of the restoration was noted by the patient and confirmed on clinical examination. A follow‐up CBCT scan obtained at 48 months after treatment showed healing of the buccal cortical bone defect that had been present at the middle third of the root before treatment (Figures 9 and 10).

**Figure 8:**
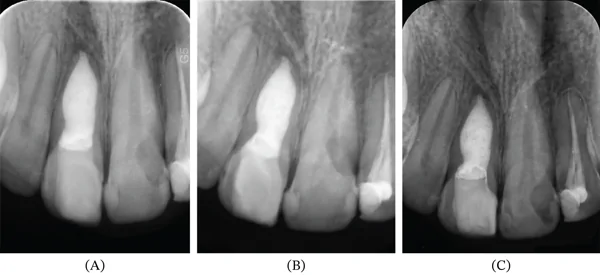
(A–C) Follow‐up periapical radiography images at 6, 12, and 48 months.

**Figure 9:**
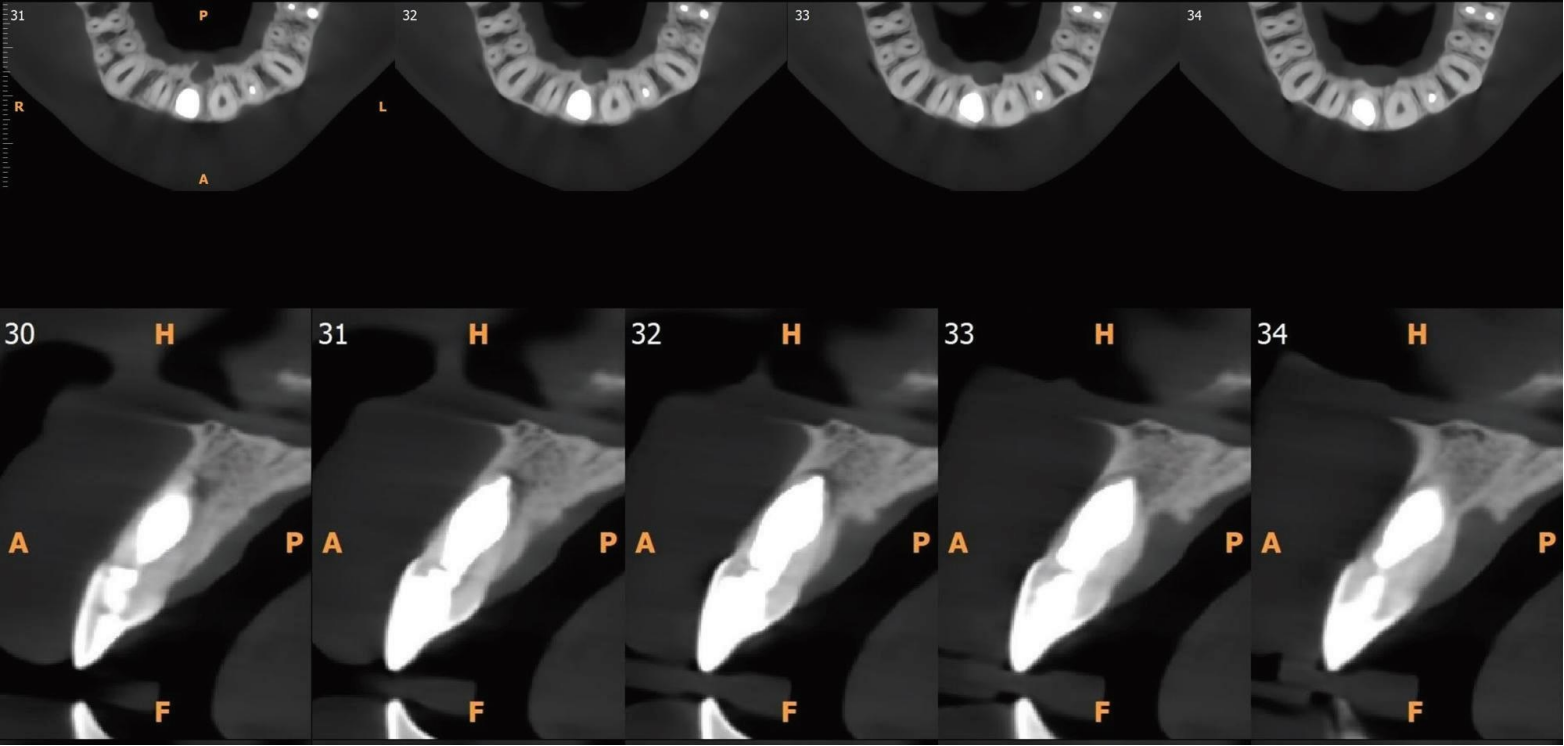
Follow‐up cross‐sectional CBCT images at 48 months after treatment show healing of the buccal cortical bone defect.

**Figure 10:**
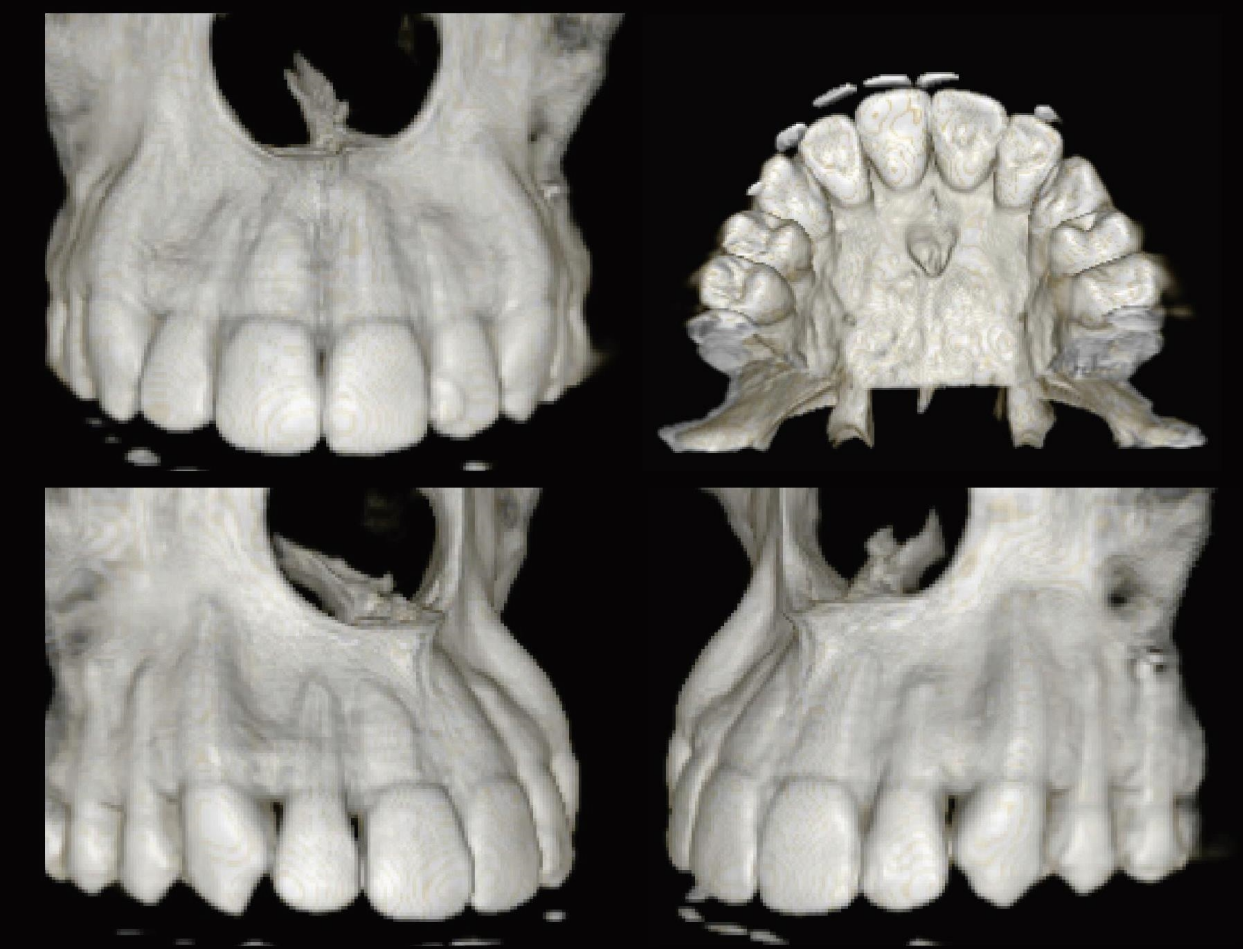
Follow‐up 3D reconstructed CBCT images at 48 months after treatment.

### 2.8. Patient Perspective

The patient reported that, before treatment, the tooth had been painful and that some of the practitioners he had consulted advised extraction and implant placement. At presentation, he described an unusual numb sensation in the tooth and felt that it was unusable. After treatment, at the 6‐month follow‐up visit, he reported no pain, mobility, or other complaints. As a young person, he was pleased that his natural tooth had been preserved, and because the tooth is in the anterior region, he was satisfied with the esthetic outcome of the tooth and the composite restoration. At the 48‐month visit, he again reported no complaints and described the tooth as asymptomatic, although he noticed a slight darkening of the restoration.

## 3. Discussion

IRR is a pathological process that begins within the pulp space and is characterized by progressive loss of dentin and dentinal tubules. This condition develops as a result of the activation of odontoclastic cells and usually affects the middle or apical third of the root canal walls. As the process progresses, the resorptive space may be filled with granulation tissue or be accompanied by metaplastic hard tissue formation [2, 12].

IRR is often asymptomatic and is detected incidentally during routine radiographic examinations. However, in the presence of active inflammation, spontaneous pain, crown tenderness, or the classic “pink tooth of Mummery” appearance may occur [2].

Although the pathogenesis of IRR is not fully understood, it is generally accepted that damage to the predentin layer and odontoblasts is necessary for the process to begin. Under normal conditions, predentin acts as a barrier that protects mineralized dentin from odontoclastic cells. Loss of this protective layer exposes the mineralized dentin surface, leading to odontoclastic activation [1, 2].

A contemporary classification of root resorption was presented in the 2023 European Society of Endodontology Position Statement on Root Resorption [13]. In this approach, resorptions are classified by jointly evaluating not only the etiology but also the anatomical localization, pathogenesis, biological behavior, and clinical features of the lesion. Thus, it is aimed to both standardize the diagnostic approach and facilitate treatment planning.

In addition to this classification, IRR lesions may also be categorized according to their perforation status as nonperforating or perforating internal resorption. In perforating lesions, progressive dentinal destruction extends through the external root surface and establishes communication with the periodontal ligament space [1]. Such cases are clinically more challenging because of the increased risk of periodontal contamination, persistent inflammation, and compromised long‐term prognosis [14]. Contemporary studies emphasize the importance of CBCT imaging for accurate assessment of perforation extent and treatment planning in these cases [15].

Current systematic reviews show that CBCT has higher sensitivity and specificity than periapical radiographs in detecting IRR. A systematic review published in 2025 reported sensitivity values for CBCT ranging from 57%–100% and specificity values ranging from 76%–100%; whereas sensitivity was lower for periapical radiographs [16].

In line with the ALARA principle, a periapical radiograph was obtained first in the present case, and a limited field‐of‐view (4 × 4 cm) CBCT scan of the anterior maxilla was added only because three‐dimensional assessment of the extent of the resorption, its buccolingual dimensions, and any root perforation was needed for treatment planning [3, 17]. A follow‐up CBCT at 48 months was obtained to reassess the buccal cortical bone defect, which cannot be evaluated on periapical radiographs.

Clinical, radiographic, and CBCT findings in this case are consistent with IRR in a nonvital right maxillary central incisor with no reported history of trauma. Pulp necrosis and chronic inflammation may have contributed, but the etiology could not be established with certainty. Trauma is the most frequently reported etiological factor in the literature, although bacterial infection, orthodontic treatment, and idiopathic causes can also play a role [1, 2]. The swelling and absence of pulp sensitivity observed before referral may indicate that pulpal infection contributed to the resorption; however, the sequence of events cannot be determined since the resorption may have preceded the symptoms, the tooth carried an old restoration and had been treated elsewhere before referral, and no histological or microbiological assessment was performed.

Early diagnosis, effective disinfection, and three‐dimensional sealing with a biocompatible material constitute the cornerstone of IRR management [9]. Surgical intervention may be considered when extensive perforation, significant loss of root structure, or difficulties in achieving adequate orthograde sealing are present. In such cases, favorable outcomes have been reported following flap surgery, apical resection, ultrasonic retrograde cavity preparation, and retrograde MTA placement [18, 19]. However, numerous studies have demonstrated complete clinical and radiographic healing of large or perforating IRR lesions after orthograde nonsurgical treatment using ultrasonically activated irrigation and bioceramic obturation, thereby avoiding the need for periapical surgery [20–22].

Sodium hypochlorite was used at every appointment for three reasons. First, the resorptive space in inflammatory internal resorption is occupied by vascularized granulation tissue or necrotic pulp remnants, and NaOCl dissolves organic tissue through its saponification and amino acid–neutralizing actions [23]. Second, resorptive cavities are irregular, enlarged spaces that rotary or hand instruments, which work in a linear manner, cannot fully contact, so chemical irrigation is essential for reaching these areas [24]. Third, NaOCl provides broad‐spectrum antimicrobial activity owing to its free chlorine content and high pH, which is important when the resorptive process is combined with pulp necrosis and bacterial contamination [25]. A 2.5% concentration was selected rather than higher concentrations to reduce the potential harm of accidental extrusion. In this case, the resorption was extensive, and although CBCT showed no perforation, the root dentin in the middle third was thin and a buccal cortical bone defect was present, so a lower concentration was preferred as a precaution against potential irrigant extrusion. This was a clinical judgment and was not compared with other concentrations. Saline was used between EDTA and NaOCl to minimize their interaction and the potential reduction in the free available chlorine of NaOCl [26].

The main reason for choosing a nonsurgical approach in this case was the accessibility of the resorptive defect via an orthograde approach and the prediction that adequate sealing could be achieved using bioceramic materials with effective disinfection [1, 10]. Owing to the CBCT evaluation, the localization of the lesion, its relationship to perforation, and the condition of the remaining dentin walls were analyzed in detail; no external root perforation or access limitations that would necessitate surgical intervention were identified [3, 17]. Furthermore, the current literature reports that orthograde treatments performed with appropriate irrigation protocols and bioceramic materials can provide successful clinical and radiographic results, even in cases of perforative IRR [20, 27].

Accordingly, a minimally invasive approach was adopted to preserve the natural tooth structure. Nonsurgical treatment offers advantages such as lower morbidity, preservation of periodontal tissues, shorter healing time, and reduced risk of surgical complications [21, 22]. In particular, irrigant activation techniques, including sonic and passive ultrasonic irrigation, may enhance the removal of debris and necrotic tissue in irregular resorptive areas, whereas bioceramic materials offer adaptation to irregular canal walls, favorable sealing properties, and biocompatibility, supporting the preference for this approach [5–7].

Therefore, in the present case, surgical intervention was reserved as a second‐line option in the event of treatment failure or persistent pathology; orthograde nonsurgical endodontic treatment was applied as the initial treatment approach.

The nonsurgical management of this nonperforating extensive IRR case, with a 48‐month follow‐up, adds a further report to the existing literature on conservative endodontic approaches [10]. Recent case reports have increasingly demonstrated favorable outcomes using advanced irrigation techniques and bioceramic materials in similar nonperforating resorptions [18, 20].

Gehlot et al. [10] reported the nonsurgical management of nonperforating IRR in a maxillary central incisor using ultrasonic irrigation and MTA repair, achieving successful clinical and radiographic outcomes with a 4‐year follow‐up. Similarly, Asgary [21] documented long‐term success with bio‐obturation in IRR cases. The present case aligns with these reports in terms of long‐term healing; however, it differs by employing EDDY sonic activation for removal of the calcium hydroxide dressing and a hybrid obturation technique consisting of MTA Angelus placed in the resorptive defect area up to the middle third, followed by an epoxy resin–based sealer and warm vertical gutta‐percha condensation in the coronal portion.

The use of MTA Angelus directly in the irregular resorptive defect was chosen because this material is reported to adapt well to irregular walls and to promote hard tissue formation, whereas the epoxy resin–based sealer was used in the remaining canal space for coronal sealing; the contribution of each material to the outcome cannot be separated in this single case. Similar hybrid approaches, which combine the bioactive properties of calcium silicate‐based materials with the sealing ability of resin‐based sealers, have been described for extensive but nonperforating resorptions [28, 29]. MTA is a calcium silicate–based material with good biocompatibility and sealing ability and the capacity to promote hard tissue formation, which makes it a suitable material for irregular resorptive cavities [7, 8].

In the present case, EDDY sonic activation was used to remove the calcium hydroxide dressing from the resorptive cavity. Recent in vitro findings indicate that sonic activation systems can achieve calcium hydroxide removal comparable with that of ultrasonic methods in irregular canal anatomies and simulated internal resorption cavities; however, a single case cannot show the specific contribution of EDDY to the clinical outcome [30, 31].

The 48‐month follow‐up period in the present case, demonstrating resolution of symptoms, no progression of resorption on periapical radiographs, and healing of the buccal cortical bone defect on follow‐up CBCT (Figures 9 and 10), is comparable with the 4‐year follow‐up reported previously for nonperforating IRR treated nonsurgically. The observed healing of the buccal cortical bone defect on follow‐up CBCT is consistent with the radiographic resolution of the periapical lesion. In this single case, the favorable long‐term outcome was achieved without surgical intervention, but the case cannot establish the effectiveness of any individual component of the protocol [10].

This report has limitations inherent to a single‐case design. There was no comparator, and the contributions of the individual steps of the protocol (NaOCl irrigation, calcium hydroxide dressing, EDTA with sonic activation, MTA, and resin‐based sealer) cannot be separated. The etiology of the resorption could not be established, and no histological or microbiological assessment was performed. The strengths of this report are the 48‐month follow‐up, the three‐dimensional CBCT documentation of the lesion before treatment and at 48 months, and the detailed description of the treatment protocol.

## 4. Conclusion

Early diagnosis and meticulous chemomechanical debridement, supported by the strategic use of bioactive materials, are generally considered central to the management of IRR. In the present case, nonsurgical orthograde treatment combined with MTA obturation was associated with healing and a stable outcome over a 48‐month follow‐up period, with no clinical symptoms or tenderness to percussion and clear radiographic evidence of periapical bone repair. Follow‐up CBCT at 48 months showed healing of the buccal cortical bone defect. CBCT played an important role in the diagnostic assessment and follow‐up, allowing precise three‐dimensional mapping of the resorptive defect that would have been more difficult to achieve with conventional periapical radiography alone. EDDY sonic activation was used for removal of the calcium hydroxide dressing in this case, but the specific contribution of this step to the outcome cannot be determined from a single case. Because resorptive lesions can behave unpredictably even after apparent resolution, continued long‐term follow‐up is essential to confirm lasting stability and to detect any recurrence of osteoclastic activity at an early stage.

## Author Contributions

Conceptualization: H.A.Y. and A.S. Data curation: H.A.Y. Formal analysis: H.A.Y. Funding acquisition: H.A.Y. and A.S. Investigation: H.A.Y. and A.S. Methodology: H.A.Y. Project administration: H.A.Y. Resources: H.A.Y. Software: H.A.Y. Supervision: H.A.Y. Validation: A.S. Visualization: A.S. Writing—original draft: H.A.Y. and A.S. Writing—review and editing: H.A.Y. and A.S.

## Funding

No funding was received for this manuscript.

## Ethics Statement

This study was reviewed and granted an official ethical exemption by the Aksaray University Rectorate Health Sciences Scientific Research Ethics Committee (Decision Date: 07/09/2026; Document Verification Code: A8EAB195‐9FE6‐47C7‐A88E‐2E6112AE86EB).

## Consent

Written informed consent was obtained from the patient to publish this report following the journal′s patient consent policy.

## Conflicts of Interest

The authors declare no conflicts of interest.

## Supporting information


**Supporting Information** Additional supporting information can be found online in the Supporting Information section. File S1: Ethical exemption certificate and CARE checklist.

## Data Availability

The data that support the findings of this study are available from the corresponding author upon reasonable request.
