# Supplementary material for: Nonsurgical Management of Extensive Internal Root Resorption: A 48‐Month Follow‐Up Case Report
Source: Case Rep Dent. 2026 Oct 22;2026:4482522. doi: 10.1155/crid/4482522 (PMC13689543; doi:10.1155/crid/4482522)
Supplement: Supplementary file 1 [file CRID-2026-4482522-s001.zip › ethical exemption certificate.pdf]

T.C.
AKSARAY ÜNİVERSİTESİ REKTÖRLÜĞÜ
Sağlık Bilimleri Bilimsel Araştırmaları Etik Kurulu

Sayı : E-19473914-050-00001250535 7.09.2026
Konu : Etik Kurul Muafiyet Talebiniz Hk

Sayın Dr. Öğr. Üyesi Hamdi Alperen YALÇIN

İlgi : 25.08.2026 tarihli başvurunuz.

Etik kurulumuza yapmış olduğunuz başvurunuz incelenmiş ve 03.09.2026 tarihindeki toplantımızda görüşülmüştür. Nonsurgical Management of Extensive Intemal Root Resorption: A 48-Month Follow-up Case Report başlıklı vaka raporu çalışmanızın Etik Kurul onayından **MUAF** olduğu değerlendirilmiştir.

Bilgilerinizi ve gereğini rica ederim.

Prof. Dr. Ayhan SARITAŞ
Kurul Başkanı

Bu belge güvenli elektronik imza ile imzalanmıştır.
Belge Doğrulama Kodu: A8EAB195-9FE6-47C7-A88E-2E6112AE86EB Doğrulama Adresi: https://www.turkiye.gov.tr/aksaray-universitesi-ebys

Adres: Aksaray Üniversitesi Bahçesaray
Telefon No: 03822883092 Faks No: 03822883099
e-Posta: İnternet Adresi: https://www.aksaray.edu.tr/
KEP Adresi:

Ayrıntılı bilgi için: Hacı Mehmet ÇELİK(SAĞ.)
Bilgisayar İşletmeni
Telefon No: 03822883092
